# Oligodendroglial Densities and Myelin Structure Are Altered in TDP‐43 Related Amyotrophic Lateral Sclerosis

**DOI:** 10.1002/glia.70090

**Published:** 2025-10-10

**Authors:** Katherine N. Lewis, Georgina A. Craig, Joel Mason, Doris Tomas, Brittany Cuic, Adam K. Walker, David G. Gonsalvez, Bradley J. Turner, Samantha K. Barton

**Affiliations:** ^1^ The Florey Institute of Neuroscience and Mental Health Melbourne Australia; ^2^ Li Ka Shing Knowledge Institute, Unity Health, St Michael's Hospital Toronto Canada; ^3^ Clem Jones Centre for Ageing Dementia Research, Queensland Brain Institute Brisbane Australia; ^4^ Sydney Pharmacy School, Faculty of Medicine and Health The University of Sydney Sydney Australia; ^5^ Department of Anatomy and Developmental Biology Monash University Melbourne Australia

**Keywords:** amyotrophic lateral sclerosis (ALS), myelin, oligodendrocyte, TDP‐43

## Abstract

Amyotrophic Lateral Sclerosis (ALS) is a fatal neurodegenerative disease characterized by the degeneration of motor neurons. However, the surrounding glia, including oligodendrocytes, also exhibit ALS pathology and TDP‐43 related dysfunction. Given that oligodendrocytes, the myelinating cells of the central nervous system, are essential for motor neuron function, they may play an underappreciated role in ALS. Here, we have extensively characterized the oligodendrocyte lineage and myelin integrity in the TDP‐43^Q331K^ mouse model of ALS. In the lumbar spinal cord of end‐stage male TDP‐43^Q331K^ mice (TDP‐43), compared to wild‐type littermates (WT), oligodendrocyte precursor cell (OPC) density, oligodendrocyte proliferation, and differentiation were all increased. There was no correlative increase in the density of mature oligodendrocytes, which was determined to be due to an increase in oligodendroglial apoptosis. In end‐stage mice, myelin reflectance was increased in the dorsal column of TDP‐43 mice, while electron microscopy showed myelin damage and misfolding in the TDP‐43 mice. Our data suggest that the oligodendrocyte lineage is impacted in TDP‐43 related ALS.

## Graphical abstract

- TDP‐43^Q331K^ mice exhibit altered proliferative dynamics in oligodendrocytes and their precursors.
- Oligodendroglial cell death is evident in TDP‐43^Q331K^ mice.
- TDP‐43^Q331K^ mice display altered myelin reflectance and pathology in the spinal cord.

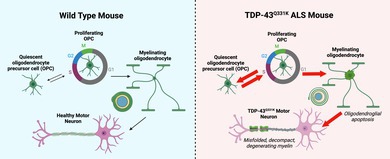

## 1 Introduction

Amyotrophic lateral sclerosis (ALS) is a rapidly progressive and ultimately fatal neurodegenerative disease, characterized by the degeneration of upper and lower motor neurons (Al‐Chalabi et al. 2016). Common to 97% of all ALS patients is the pathological accumulation of TDP‐43 protein, an essential DNA and RNA binding protein required for transcription, splicing, and processing of RNA, maintaining mRNA stability, and RNA metabolism through communicating bi‐directionally between the cytoplasm and nucleus (Ayala et al. 2006; Dong and Chen 2018; Tollervey et al. 2011). Despite the ubiquitous expression of TDP‐43, oligodendrocytes, in the context of TDP‐43 related ALS, remain largely understudied, despite pathological TDP‐43 aggregates being identified in these myelinating cells (Barton, Magnani, et al. 2021; Heo et al. 2022; Neumann et al. 2007). Given the crucial role of oligodendrocytes in supporting motor neuron health and function (Sherman and Brophy 2005), there is clearly a critical need to ascertain the contributions of oligodendrocytes to ALS in a TDP‐43 context.

Oligodendrocytes have been characterized in the SOD1^G93A^ mouse model of ALS. Even before symptom onset, increased proliferation of oligodendrocyte precursor cells (OPCs) was observed, and subsequently an increased density of OPCs in the lumbar spinal cord of the SOD1^G93A^ mice; yet there was no overall change to the density of mature oligodendrocytes, which was attributed to oligodendrocyte death (Kang et al. 2013; Philips et al. 2013). Myelin perturbations were also observed in the ventral horn gray matter in the SOD1^G93A^ mice but were not present in healthy littermates (Kang et al. 2013). In the context of TDP‐43, the selective deletion of *TARDBP* (the gene that encodes the TDP‐43 protein) from PDGFRα^+^ OPCs resulted in almost complete loss of OPCs from the corpus callosum (Heo et al. 2022), while the removal of *TARDBP* from MOBP^+^ premyelinating oligodendrocytes in mice led to seizures and premature death (Heo et al. 2022). When the *TARDBP* gene was excised from mature oligodendrocytes, the knockout mice exhibited reduced grip strength by 7 weeks of age, coordination and motor deficits, seizures and premature death by postnatal day 90, and a rise in OPC proliferation in the spinal cord gray matter (Wang et al. 2018). Thus, studies in selective TDP‐43 knock‐out mice, coupled with the changes observed in SOD1‐related ALS mice, suggest a pivotal role of the oligodendrocyte lineage in ALS. Indeed, induced overexpression of the ALS‐linked TDP‐43^M337V^ in oligodendrocytes alone using an MBP‐cre caused motor deficits in mice, downregulation of myelinating oligodendrocyte RNA pathways, and increased oligodendrocyte death (Horiuchi et al. 2024).

In the present study, we have extensively characterized the oligodendrocyte lineage and myelin reflectance and appearance throughout the disease course in the *TDP‐43* ^*Q331K*^ mouse model of ALS. We observed increased OPC proliferation and differentiation, and abnormal myelin in the *TDP‐43* ^*Q331K*^ mice, suggesting that oligodendroglia are impacted in TDP‐43 relevant ALS. Given the pivotal role of oligodendrocytes in supporting motor neuron health and function, perturbations to oligodendrocyte function may contribute to motor neuron decline, dysfunction, and loss in ALS.

## 2 Materials and Methods

### 2.1 Transgenic TDP‐43^Q331K^ Mouse Model of ALS

TDP‐43Q331K (B6N.Cg‐Tg(Prnp‐TARDBP*Q331K)103Dwc/J, high expressing line 103, stock number 017933) mice were purchased from the Jackson Laboratory (Bar Harbor, ME) and maintained on a C57BL/6NJ background.

The *TDP‐43* ^*Q331K*^ transgenic mouse drives human *TARDBP* with a pathogenic glutamine to lysine substitution at amino acid position 331 (Q331K), driven by the mouse prion promotor (Arnold et al. 2013). Male TDP‐43^Q331K^ heterozygous mice (TDP‐43; *n* = 3–6 per time point), and their wild‐type male littermates (WT; *n* = 3–6) were collected at P15, P30, 3, 5, 8, and 10 months for analysis. Due to a weight gain phenotype in female *TDP‐43* ^*Q331K*^ mice, also noted previously (Mitchell et al. 2015), only male mice were used for this study. The *TDP‐43* ^*Q331K*^ mouse has been extensively characterized; it exhibits symptom onset from 2 months, including motor neuron loss and motor behavior deficits, and phenotypes do not worsen after 10 months (Arnold et al. 2013). In our mice, there were no differences in the weights of male mice (Figure S1A). We mirrored previous behavioral phenotypes identified using this mouse model with decreased performance on the rotarod motor test (Figure S1B,C) and decreased hind‐limb grip strength in 10‐month‐old TDP‐43 mice (Figure S1D,E).

### 2.2 BrdU and EdU Administration

For cell tracking through double S‐phase labelling (Gonsalvez et al. 2013) in mice aged P15, P30, 3, and 5 months, intraperitoneal (IP) injection of BrdU (B5002, Sigma; 100 μg/g) was administered. After 2 h, an IP injection of EdU (E10415, Thermo; 50 μg/g) was administered. Mice were sacrificed 30 min post‐EdU injection via lethal overdose (sodium pentobarbitone, 100 mg/kg; IP).

### 2.3 EdU Administration for 8 and 10 Months Animals

Mice collected at 8 and 10 months were administered EdU in their drinking water at 0.2 mg/mL for 7 days. This change in protocol in latter age groups was chosen given that the cell cycle length decreases with age (Psachoulia et al. 2009) so the short duration of double S‐phase labelling would not result in sufficient labelling. The EdU was replaced every 2–3 days, prior to euthanasia via lethal overdose (sodium pentobarbitone, 100 mg/kg; IP). Given the change in EdU labelling methodologies between the younger (P15, P30, 1‐, 3‐, 5‐month‐old mice) and older (8‐ and 10‐month‐old mice), the data obtained for the different labelling methods were not directly compared. While EdU can sufficiently track cells that have undergone proliferation, then differentiation, it will not label cells that differentiate from their quiescent state (Hughes et al. 2013); thus, there may be some new oligodendrocytes not labeled with EdU.

### 2.4 Tissue Collection and Processing

Mice were transcardially perfused with 0.1 M phosphate buffered saline (PBS), followed by 4% paraformaldehyde (PFA) in 0.1 M phosphate buffer (PB), and the spinal cord was removed. Tissue was left in 4% PFA overnight at 4°C, washed with 3 × 0.1 M PBS, then transferred into 30% sucrose in PBS. Lumbar spinal cord was dissected, then halved transversally. Sections were frozen in O.C.T (SciGen). The rostral block was serially sectioned (25 μm) transversally in sets of 6–12 slides, 10–12 sections per slide for immunocytochemistry and Ventral Horn Gray Matter (VHGM) SCoRe microscopy. For SCoRe microscopy of the dorsal column (DC), the caudal half of the lumbar spinal cord was serially sectioned longitudinally (20 μm) in sets of 3–6 slides, 8–10 sections per slide. For EM, lumbar spinal cord tissue was postfixed in Karnovsky buffer (2% glutaraldehyde and 2% PFA in 0.1 M sodium cacodylate) before being washed 3× with 0.1 M sodium cacodylate, then stored at 4°C in 0.1 M sodium cacodylate until processing. Processing occurred in collaboration with the Peter MacCallum Cancer Centre, who postfixed samples in 2% OsO4, then transferred samples through a series of concentrated ethanol stages (50%, 70%, 90%, 100%), to acetone, and finally from diluted (1 resin:3 acetone, 1 resin:1 acetone, 3 resin:1 acetone) to 100% resin. Semi‐thins (0.5 μm) were cut to confirm correct orientation, followed by ultra‐thin sections (90 nm) contrasted using uranyl acetate and lead citrate.

### 2.5 Immunohistochemistry

One slide was stained per antibody. O.C.T was removed using 0.1 M PBS. Primary antibodies (BrdU rat, Abcam, 1:100; Ki67 rabbit, ThermoFisher, 1:50; Ki67 rat, ThermoFisher, 1:50; Olig2 rabbit, Abcam, 1:200; PDGFRα goat, Abcam, 1:100; CC1 mouse, 1:1000; Caspase‐3 mouse, Invitrogen, 1:30; Parvalbumin rabbit, ThermoFisher, 1:500) were incubated for 24–48 h in 10% NDS in 0.3% TritonX‐PBS (or 10% NDS in PBS for slides utilized for SCoRe imaging). For BrdU staining, slides were immersed in 2 M Hydrochloric Acid for 30 min for antigen retrieval, then washed 2 × 10 min in 0.1 M sodium tetraborate to neutralize the acid. For Caspase‐3 and parvalbumin antigen retrieval, slides were immersed in 8 M EDTA at 95°C for 10 min, followed by a 5 min PBS wash. Secondary antibodies (all raised in Donkey and used at 1:200; Jackson Immuno Research) were incubated alongside Hoechst (Sigma, 1:1000) for 1.5–2 h. EdU was detected via application of a Click‐it Alexa 647 kit (ThermoFisher, C10340), prepared according to the manufacturer's protocol. A TUNEL Assay Kit (BrdU Red ab66110) was used as per manufacturer's instructions. Slides were coverslipped using Fluorescent Mounting media (DAKO).

### 2.6 Imaging and Cell Counts

Imaging was performed on the LSM780 Zeiss Confocal Microscope (Carl Zeiss Inc.) and LSM900 Zeiss Confocal Microscope (Carl Zeiss Inc.) using a 20× air lens. 3–6 sections were examined per mouse, and 3–5 mice were analyzed per metric; specific n detailed in each figure. The experimenter was blinded throughout. Tiled images of the lumbar spinal cord encompassed the DC, corticospinal tract (CST), VHGM, and ventral horn white matter (VHWM). Alternating hemispheres were analyzed on each section. Images were analyzed in FIJI Image J software via the cell counter plug‐in.

### 2.7 Spectral Confocal Reflectance Microscopy (SCoRe)

SCoRe is an imaging technique that quantifies myelin density, due to the unique refractive index of compact myelin (Schain et al. 2014). Three confocal lasers (458, 561, and 633 nm) are utilized to collect reflection signals and combined to represent whole myelinated axons. All SCoRe images were taken with the LSM780 Zeiss Confocal Microscope (Carl Zeiss Inc.) using a 40× oil lens. For each age group, a threshold was selected that best captured bona fide myelin processes but reduced background noise and was maintained for all images. The percentage of positive pixels for the tissue area of interest was analyzed using FIJI on 3–12 images per mouse, 3–4 mice per group.

### 2.8 Transmission Electron Microscopy (TEM)

Imaging was performed using the JEOL 1400 flash. Three male mice per group were assessed, with 6–10 images obtained of the DC of the lumbar spinal cord at 5000× magnification.

### 2.9 Cell Cycle Calculations

Using a formula adapted from previous studies (Hayes and Nowakowski 2000), the growth fraction can be calculated as:

$$\mathrm{GF}=\frac{\mathrm{Ki}67^{+}{\text{PDGFRα}}^{+}\text{cells}}{{\text{PDGFRα}}^{+}\text{cells}}\times 100\%$$

Assuming a steady state of OPC cell cycling (Gonsalvez et al. 2013; Hayes and Nowakowski 2000), a combination of BrdU and EdU tracing were used to calculate S‐phase length (*T*_s_) and cell cycle length (*T*_c_) as per the following equations (whereby LI_0_ equates to the proportion of cells in the S‐phase at a single moment):

$$T_{s}=2\times \frac{{\text{BrdU}}^{+}{\text{PDGFRα}}^{+}\text{cells}}{{\text{BrdU}}^{+}{\mathrm{EdU}}^{-}\;{\text{PDGFRα}}^{+}\text{cells}}$$

$$T_{c}=\frac{T_{s}\times \mathrm{GF}}{{\mathrm{LI}}_{0}}$$

When LI_2.5_ and LI_0.5_ were plotted linearly as per the equations below; the y‐intercept equates to LI_0_.

$${\mathrm{LI}}_{2.5}=\frac{{\text{BrdU}}^{+}{\text{PDGFRα}}^{+}\text{cells}}{{\text{PDGFRα}}^{+}\text{cells}}\times 100$$

$${\mathrm{LI}}_{0.5}=\frac{{\mathrm{EdU}}^{+}{\text{PDGFRα}}^{+}\text{cells}}{{\text{PDGFRα}}^{+}\text{cells}}\times 100$$

### 2.10 Statistical Analysis

All data are expressed as the mean ± the standard error of the mean (SEM). All data were analyzed using GraphPad Prism software. Cell densities and SCoRe were assessed using 2‐way ANOVA with Tukey's multiple comparisons post hoc test, while g‐ratio was assayed via multiple unpaired *t* tests, and TUNEL, caspase‐3, and TEM myelin pathologies were compared via Student's unpaired *t* tests. A statistically significant difference was concluded if *p* < 0.05.

## 3 Results

### 3.1 OPCs Exhibit Increased Proliferation and Differentiation in TDP‐43 Mice

**FIGURE 1:**
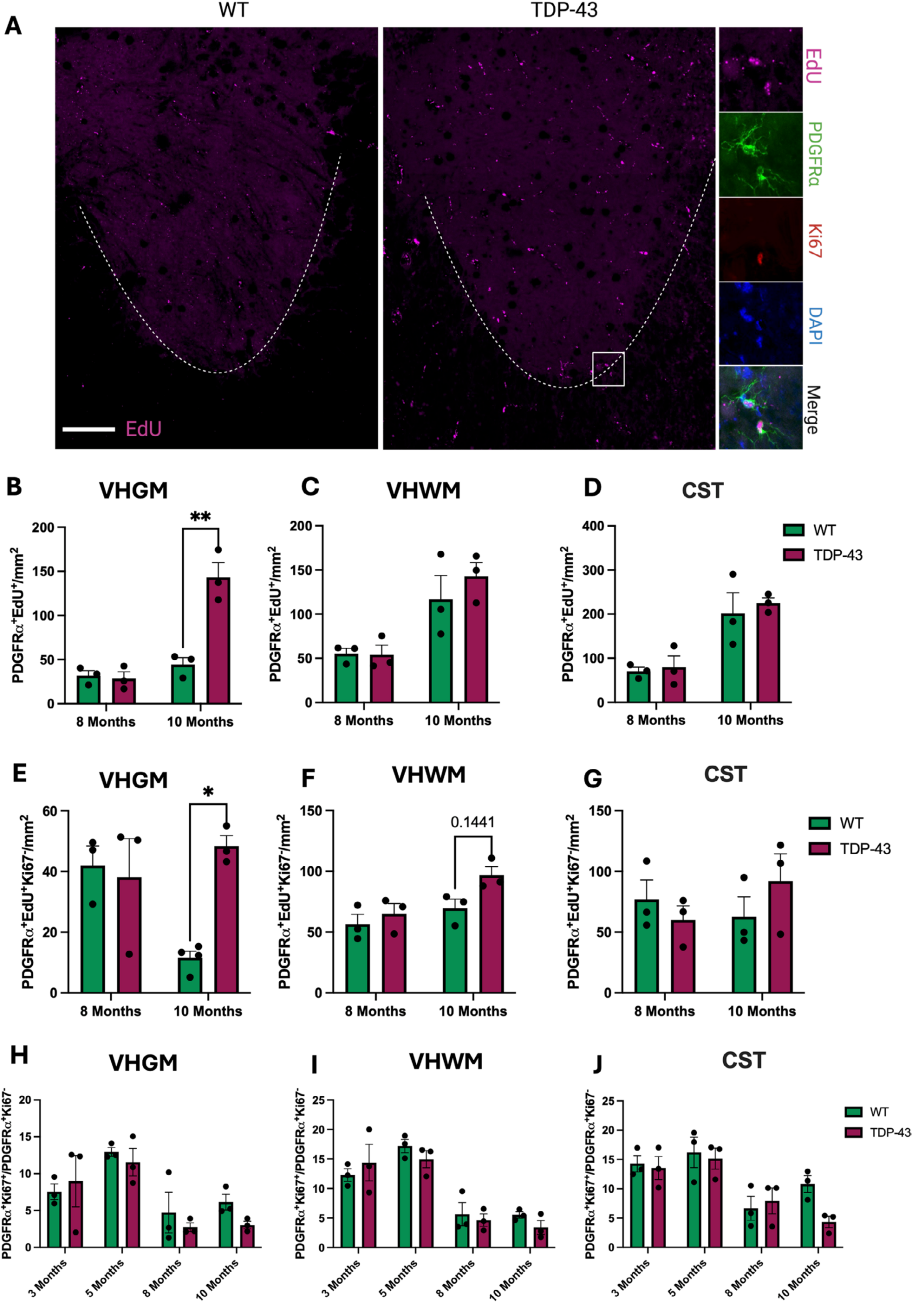
Proliferation of oligodendrocyte precursor cells (OPCs) over 7 days with EdU administration. (A) Representative image of a TDP‐43 mouse lumbar spinal cord section compared to WT, scale bar 200 μm. OPCs are marked by PDGFR⍺ (green), cells that had proliferated in the 7 days prior to culling are marked by EdU (magenta), cells that were in the proliferative state at the time of death are marked by Ki67 (red), and all nucleated cells are marked by DAPI (blue). (B) There were significantly more proliferating OPCs in the ventral horn gray matter (VHGM) of TDP‐43 mice at 10 months, compared to WT (2‐way ANOVA *p* = 0.0016; post hoc at 10 months *p* = 0.0057). In the ventral horn white matter (VHWM; C) and corticospinal tract (CST; D) there were no changes in OPC proliferation at either age. At 10 months, VHGM OPCs in the TDP‐43 mice showed an increase quiescent OPCs compared to WT (E; 2‐way ANOVA *p* = 0.0391; post hoc at 10 months *p* = 0.0148). Again, in the VHWM (F) and the CST (G) there were no significant changes in OPC proliferative behavior at either age. OPC growth fraction, that is, the density of dividing cells as a ratio of all cells, was not changed in the VHGM (H), VHWM (I), or CST (J) at any age or by genotype. All data are presented as mean ± S.E.M, *p < 0.05, **p < 0.01; 2‐way ANOVA with Tukey's post hoc test; *n* = 3 per genotype per age.

In 8‐ and 10‐month‐old mice, which received EdU for 7 days in drinking water, PDGFR⍺^+^ OPCs proliferation status was distinguished by EdU and Ki67 labelling; PDGFR⍺^+^EdU^+^Ki67^+^ cells were considered currently dividing OPCs, while PDGFR⍺^+^EdU^+^Ki67^−^ cells constituted newly generated OPCs that had returned to quiescent OPCs and not differentiated (Figure 1A). The overall density of newly generated OPCs was significantly increased in the VHGM at 10 months in the TDP‐43 mice compared to WT (Figure 1B; 2‐way ANOVA *p* = 0.0016; post hoc at 10 months *p* = 0.0057), but no changes were observed at 8 months, nor in the VHWM (Figure 1C) or CST (Figure 1D). There was also a significant increase in the density of OPCs that had proliferated and returned to quiescence in the VHGM at 10 months (Figure 1E; 2‐way ANOVA *p* = 0.0391; post hoc at 10 months *p* = 0.0148), but no changes at 8 months nor in other regions (Figure 1F,G). In mice spanning P15 through to 5 months, where mice underwent double S‐phase labelling, there were no significant changes to OPC S‐phase length or cell cycle length in the DC, CST, VHGM, or VHWM of the lumbar spinal cord (Figure S2). The OPC growth fraction (proportion of PDGFR⍺^+^Ki67^+^ proliferating OPCs to nonproliferating PDGFR⍺^+^Ki67^−^) was not different at any time point between WT and TDP‐43 in the VHGM (Figure 1H), VHWM (Figure 1I), CST (Figure 1J), or DC (Figure S3B).

**FIGURE 2:**
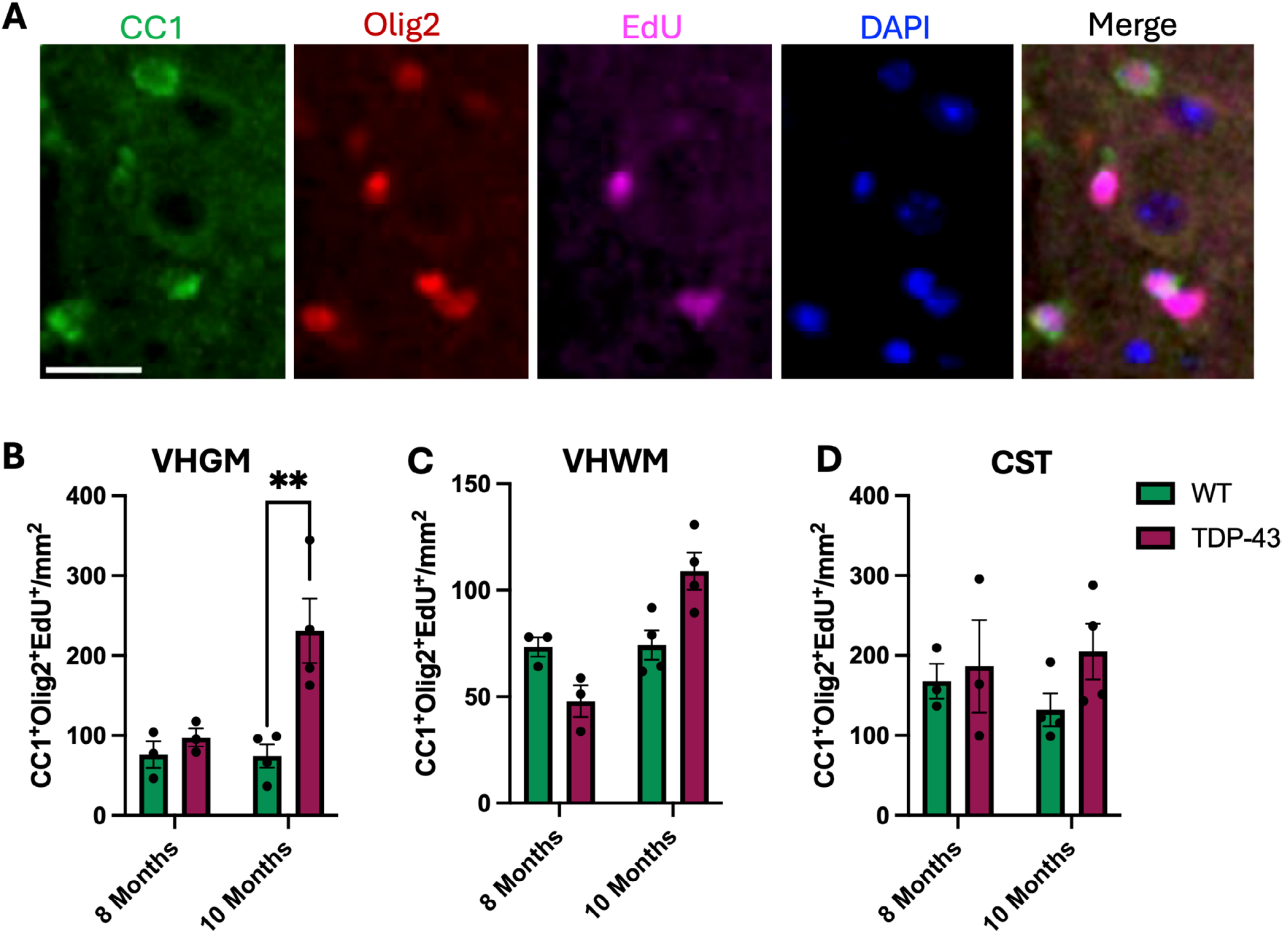
The differentiation of OPCs to matured oligodendroglia over 7 days of EdU administration. (A) Representative image of a TDP‐43 mouse lumbar spinal cord section, scale bar 20 μm. Matured oligodendroglia are marked by CC1 (green), cells that have proliferated in the 7 days prior to culling are marked by EdU (magenta), all oligodendroglia are marked by Olig2 (red) and all nucleated cells are marked by DAPI (blue). Cells that are CC1^+^Olig2^+^EdU^+^ are considered differentiated oligodendroglia. In TDP‐43 mice at 10 months the density of differentiated oligodendroglia was increased in the ventral horn gray matter (VHGM) of TDP‐43 mice compared to WT (B; 2‐way ANOVA *p* = 0.0077; post hoc at 10 months *p* = 0.0056). There were no changes in the ventral horn white matter (VHWM; C) or corticospinal tract (CST; D) at either age. All data are presented as mean ± S.E.M, *p ≤ 0.05, **p ≤ 0.01; 2‐way ANOVA with Tukey's post hoc test; *n* = 3–4 per genotype, per age.

To assess oligodendrocyte differentiation, the density of CC1^+^Olig2^+^EdU^+^ oligodendrocytes was quantified (Figure 2A). Differentiation was significantly increased in TDP‐43 compared to WT at 10 months in the VHGM (Figure 2B; 2‐way ANOVA *p* = 0.0077; post hoc at 10 months *p* = 0.0056). There was no change at other time points or in the VHWM (Figure 2C) or CST (Figure 2D).

### 3.2 OPC, But Not Oligodendrocyte, Densities Are Increased in TDP‐43 Mice

**FIGURE 3:**
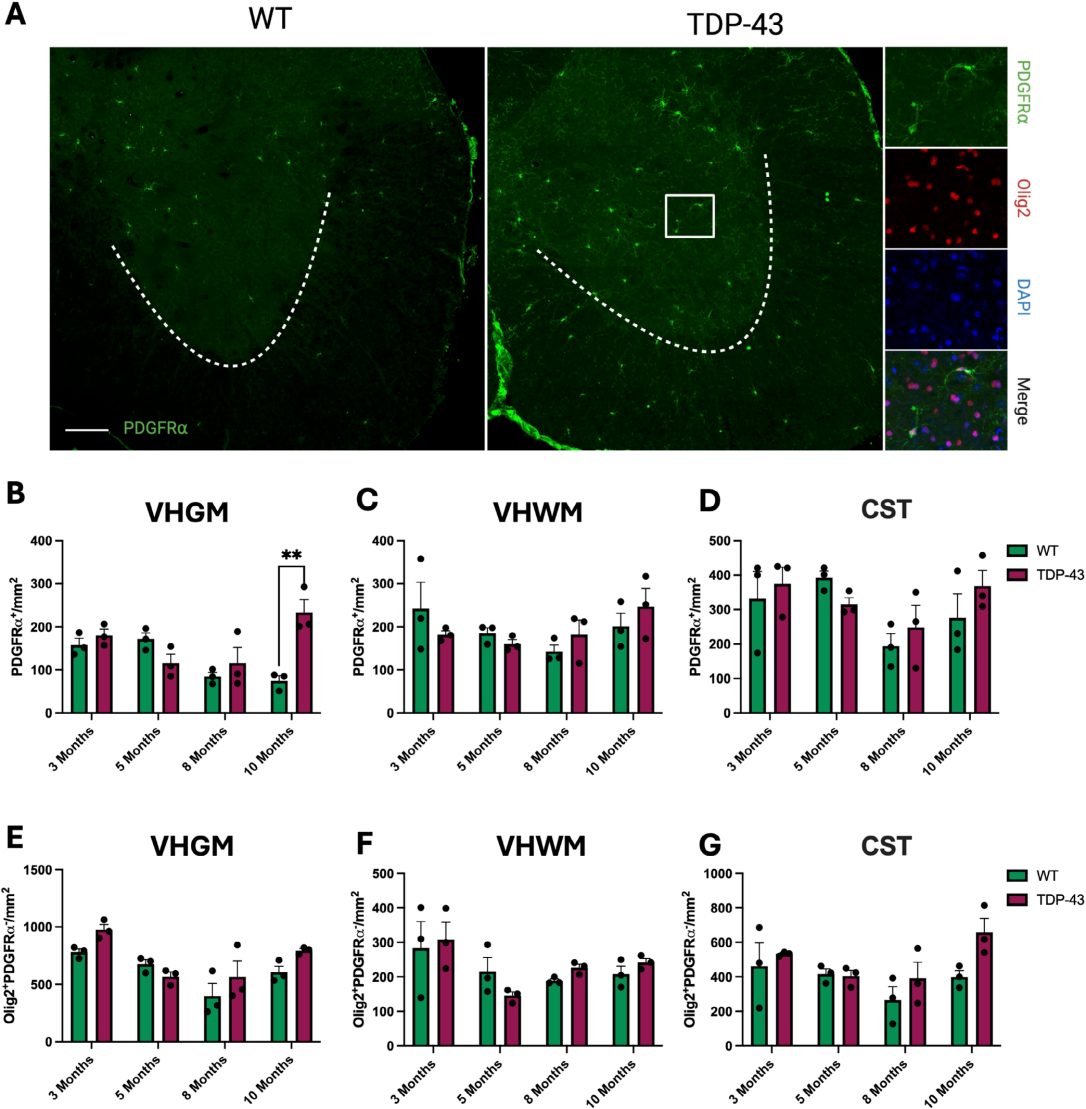
OPC and oligodendroglial densities TDP‐43 mice compared to WT. (A) Representative images of labeled oligodendrocyte precursor cells (OPCs; PDGFR⍺; green) and matured oligodendrocytes (Olig2; red). DAPI (blue) labels all nucleated cells, scale bar 200 μm. (B) OPC density (PDGFR⍺^+^ cells) was significantly increased in the ventral horn gray matter (VHGM) of 10‐month‐old TDP‐43 mice when compared to WT (2‐way ANOVA *p* = 0.0181; post hoc at 10 months *p* = 0.0014) and was not altered in the ventral white matter (VHWM; C) or corticospinal tract (CST; D) at any age. Matured oligodendroglial density was not changed in the VHGM (E), VHWM (F), or CST (G) at any age or by genotype. All data are presented as mean ± S.E.M, **p ≤ 0.01; 2‐way ANOVA with Tukey's post hoc test; *n* = 3 per genotype per age.

We next characterized whether the increased proliferation and differentiation of OPCs in end‐stage TDP‐43 mice altered the overall densities of OPCs (Olig2^+^PDGFR⍺^+^) and oligodendrocytes (Olig2^+^PDGFR⍺^−^; Figure 3A). OPC density was significantly increased at 10 months in TDP‐43 compared to WT in the VHGM (Figure 3B; 2‐way ANOVA *p* = 0.0181; post hoc at 10 months *p* = 0.0014), but not at other time points nor in other regions (Figure 3C,D and Figure S3A,B). The density of mature oligodendrocytes was not different at any time point in the VHGM (Figure 3E), VHWM (Figure 3F), and CST (Figure 3G).

**FIGURE 4:**
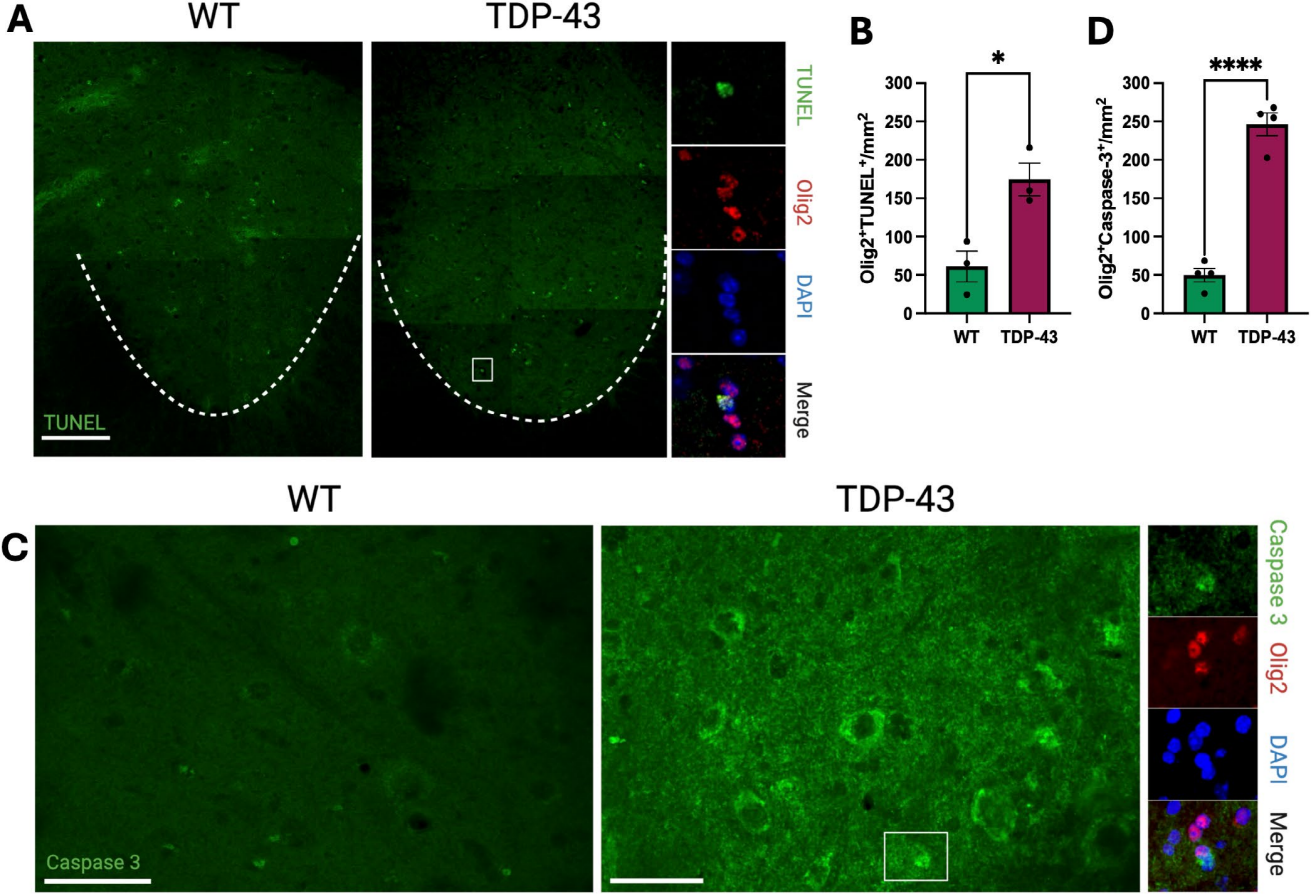
Ten‐month‐old TDP‐43 mice exhibit increased oligodendroglial death compared to WT. (A) Representative images of labeled oligodendroglia (Olig2; red) and apoptotic cells (terminal deoxynucleotidyl transferase biotin‐dUTP nick end labelling, TUNEL; green) in the ventral horn gray matter (VHGM) of the lumbar spinal cord, scale bar 200 μm. (B) The density of TUNEL labeled oligodendroglia was significantly increased in the TDP‐43 mice compared to WT (*p* = 0.018). (C) Representative images of caspase‐3 labeled cells (green) in the VHGM of 10‐month‐old WT and TDP‐43 mice, scale bar 200 μm. (D) The density of caspase‐3 labeled oligodendroglia was significantly increased in VHGM TDP‐43 mice compared to WT (*p* ≤ 0.0001). Data presented as mean ± S.E.M, *p ≤ 0.05, ****p ≤ 0.0001, student's unpaired *t* tests; *n* = 3–4 mice per genotype.

Due to the increase in OPC proliferation and differentiation in 10‐month‐old TDP‐43 mice compared to WT, yet no increase in the density of mature oligodendrocytes, we conducted a terminal deoxynucleotidyl transferase biotin‐dUTP nick end labeling (TUNEL; BrdU‐Red) assay, which detects fragmented DNA in apoptotic cells. When co‐labeled with Olig2 (Figure 4A), 10 months TDP‐43 mice exhibited increased oligodendroglial cell death compared to WT in the VHGM region (Figure 4B; *p* = 0.018). When Olig2+ oligodendrocytes were co‐labeled with caspase‐3, which also labels apoptotic cells, the same increase was noted. At 10 months, TDP‐43 mice exhibited increased caspase‐3 mediated oligodendrocyte cell death in the VHGM compared to WT (*p* < 0.0001; Figure 4C).

### 3.3 Myelin Dysfunction Is Abundant in End‐Stage TDP‐43 Mice

**FIGURE 5:**
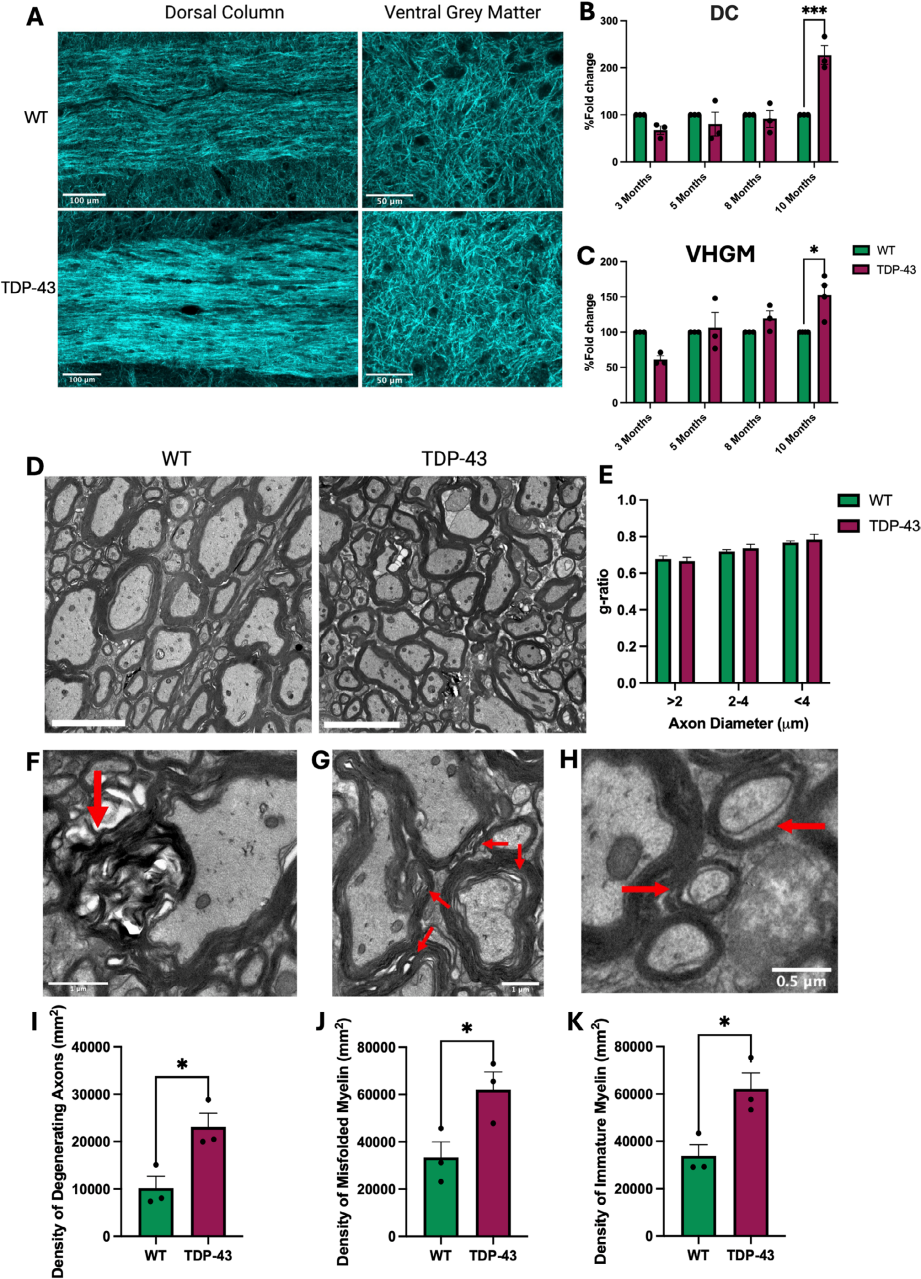
TDP‐43 mice exhibit altered myelin characteristics. (A) Representative Spectral Confocal Reflectance (SCoRe) microscope images of lumbar spinal cord dorsal column (DC) and ventral horn grey matter (VHGM) in 10‐month‐old mice. (B) There was a significant increase in myelin reflectance (the % of positive pixels of the dense white matter tract, represented as a fold change to WT) in TDP‐43 mice compared to WT in the dorsal column (DC; 2‐way ANOVA *p* = 0.0094; post hoc at 10 months *p* ≤ 0.001) and in the VHGM (C; 2‐way ANOVA *p* = 0.0184; post hoc at 10 months *p* = 0.013) only at 10 months. (D) Representative image of Transmission Electron Micrographs (TEM), which was conducted on 10‐month‐old mice in the DC, scale bar 5 μm. (E) There were no differences in the g‐ratios of myelinated axons between WT and TDP‐43 mice. Electron micrographs showed axonal degeneration (F; red arrows), misfolded myelin (G; red arrows), and axons with immature myelin (H; red arrows), all of which were significantly increased in 10‐month‐old TDP‐43 mice (I, *p* = 0.023; J, *p* = 0.011; K, *p* = 0.027). All data are presented as mean ± S.E.M, *p ≤ 0.05; **p ≤ 0.01; ***p ≤ 0.001; SCoRe data conducted via 2‐way ANOVA with Tukey's post hoc test; g‐ratios analyzed via multiple unpaired *t* tests; *n* = 3–4 per genotype per age.

To determine if myelin density is altered in TDP‐43 mediated ALS, we utilized SCoRe (Figure 5A), which uses reflected laser light to provide a quantitative measure of myelin reflectance, which can be a surrogate marker for myelin density. SCoRe analysis of the DC revealed a significant increase in myelin reflectance at 10 months in the TDP‐43 mice compared to WT (Figure 5B; 2‐way ANOVA *p* = 0.0094; post hoc at 10 months *p* < 0.001) but no changes at any other time point. Similarly, in the VHGM of 10‐month‐old TDP‐43 mice there was a significant increase in myelin reflectance (Figure 5C; 2‐way ANOVA *p* = 0.0184; post hoc at 10 months *p* = 0.013) but no changes at any other age. Since VHGM myelin would likely be ensheathing interneurons, which may be themselves pathological, we investigated and identified caspase‐3 activation within parvalbumin^+^ interneurons in the VHGM at 10 months (Figure S5). We also employed TEM of cross‐sections of the DC of 10‐month‐old TDP‐43 and WT mice (Figure 5D) and found no differences in the g‐ratios of myelinated axons (Figure 5E) nor in the density of myelinated fibers between the TDP‐43 and WT (Figure S6). Myelin phenotypes such as degenerating axons (Figure 5F), misfolded myelin (Figure 5G), and immature myelin, as identified by noncompact myelin loops (Figure 5H), were also examined and were characterized according to the methodology of previous studies (Kang et al. 2013; Li et al. 2018; Trapp et al. 1982). In 10‐month‐old TDP‐43 mice there was a significant increase in the densities of degenerating axons (Figure 5I; *p* = 0.023), axons ensheathed with misfolded myelin (Figure 5J; *p* = 0.011), and axons ensheathed with immature myelin (Figure 5K; *p* = 0.027). Taken together, these data show that while more myelin may be being produced, as evidenced by increased immature myelin, the myelin itself is not compact, misfolded, and/or degenerating.

## 4 Discussion

Oligodendrocytes and their precursors have a plethora of functions that are integral to brain homeostasis: not only do they receive and send signals that modulate their cellular environment (Thornton and Hughes 2020), but they are also essential for myelination and maintaining neuronal health (Sherman and Brophy 2005). Given the pivotal roles pertaining to supporting neuronal function, it highlights the need to understand the contributing role of oligodendrocytes in ALS. This study is the first to characterize the entire oligodendrocyte lineage in a TDP‐43 relevant ALS mouse model. In TDP‐43^Q331K^ mice, OPC proliferation and differentiation were significantly increased; however, the population of postmitotic oligodendroglia did not alter between TDP‐43 and WT mice, which can be attributed to an increase in oligodendroglial death in the TDP‐43 mice. Myelin was also affected in the TDP‐43 mice, with higher densities of degenerating, misfolded, and immature myelin surrounding axons. This combination of pathologies highlights key abnormalities in the maintenance of the oligodendrocyte lineage and their myelin production in ALS.

In contrast to studies that display a complete loss of function in oligodendrocytes alone, the TDP‐43^Q331K^ mouse used in the present study exhibits a mild over‐expression of human mutant TDP‐43 throughout the entire nervous system (Arnold et al. 2013). Despite this contrast, we also found overlapping phenotypes, including OPC proliferation, oligodendroglial death, and myelin deficits, suggesting that both the loss and gain of function pathologies of TDP‐43 proteinopathy have significant and detrimental impacts on oligodendroglia and myelin. It would be interesting to further stratify the *TDP‐43* ^*Q331K*^ mutation by cell type to ascertain whether the changes we identify are intrinsic to the oligodendrocyte or secondary to neuronal changes. Interestingly, in the *TDP‐43* ^*Q331K*^ mice utilized for this study, the most severe motor behavior pathology was identified at 10 months, aligning with the changes observed in the oligodendroglial population, highlighting that the pathology of the oligodendroglia may be exacerbating motor neuron dysfunction, particularly influencing the lower motor neurons that control motor performance. Indeed, the changes we observed in the oligodendroglial lineage were region specific, with the majority of changes found in the VHGM of the lumbar spinal cord, thereby implicating a role of the lower motor neuron cell bodies, the interneuronal cell population, or other spinal glial cell types. Oligodendroglia dysfunction in the VHGM was also identified in *SOD1* ^*G93A*^ mice (Kang et al. 2013; Philips et al. 2013), cementing the implication of these cells in ALS. Reactive oxygen species, as a result of excitotoxicity, have been suggested to propagate from motor neuron somas to astrocytes in both SOD1 mouse models and human patients (Rao and Weiss 2004), while endoplasmic reticulum stress from motor neurons can propagate toxicity to astrocytes and oligodendrocytes (Sun et al. 2015), thus motor neurons may indeed be propagating their pathology to the surrounding oligodendroglia. However, given we also observed an increase in myelin reflectance and caspase‐3 activation in the VHGM, it could be spinal interneurons driving the oligodendroglial changes, as the interneuronal myelin is more prevalent in this region. While less commonly studied in ALS, spinal interneurons do exhibit neurodegeneration and cell loss in a similar manner to motor neurons in ALS patient postmortem samples (Stephens et al. 2006) and TDP‐43 related mouse models of ALS (Reale et al. 2023; Tsuboguchi et al. 2023). Given the interneurons that are myelinated are parvalbumin‐positive (Veshchitskii and Merkulyeva 2023) and are highly abundant in the VHGM, it would be interesting to interrogate this population. Ultimately, our data highlight the need to determine whether preserving oligodendrocyte function could preserve neuronal function. Recent data have shown that in ALS patients with high levels of cytoplasmic phosphorylated TDP‐43, myelin genes were downregulated as a direct consequence (Wang et al. 2025); however, perhaps upregulating promyelinating pathways can provide a therapeutic avenue.

The concept that perturbed TDP‐43 may impact oligodendrocyte function is unsurprising. As an RNA‐binding protein, TDP‐43 is known to bind to myelin genes such as myelin regulatory factor (Zhu et al. 2024), sterol regulatory element‐binding factor 2, a cholesterol gene that also regulates myelination (Ho et al. 2021), and heterogeneous nuclear ribonucleoprotein A1 and A2/B1, which are involved in trafficking essential myelin proteins within oligodendrocytes (Barton et al. 2019; Torvund‐Jensen et al. 2014; White et al. 2008), so perturbations to TDP‐43 function would likely impact myelin. Indeed, in both sporadic and familial ALS human postmortem tissue, it was identified that trafficking of myelin‐associated RNAs was impaired (Barton, Gregory, et al. 2021). Further, when Heo and colleagues selectively deleted *TDP‐43* from MOBP^+^ premyelinating oligodendrocytes, they found that the oligodendrocytes inappropriately wrapped myelin around axonal cell bodies and nonneuronal structures (Heo et al. 2022). This is a phenotype also observed in demyelination studies, whereby existing oligodendrocytes attempting to re‐myelinate neurons exhibit increased mistargeting compared to newborn oligodendrocytes (Neely et al. 2022). With respect to myelin integrity, when *TDP‐43* was deleted from either OPCs or mature oligodendrocytes, analyses of EM imaging showed myelin was decreased in overall density and was thinner (Wang et al. 2018). In contrast, in the current study there was increased myelin reflectance, which can imply increased myelin density, but our EM analyses suggest there was no change in myelin thickness and, instead, show structurally aberrant myelin. It is important to note that myelin debris from degrading myelin sheaths can also emit reflection picked up by SCoRe microscopy (Gonsalvez et al. 2019). Thus, while increased reflectance could be due to *de novo* oligodendroglial production resulting in new myelin to protect against axon loss, the overall increase in myelin reflectance may also be attributed to myelin debris, swelling, and damage. Moreover, while increasing the production of myelin may be a desirable concept to combat myelin loss, hypermyelination does not improve motor neuron function in ALS models (Fusco et al. 2019; Hernández et al. 2023); thus, promoting excess myelination may not be an appropriate therapeutic target for treating ALS. Nevertheless, understanding how myelin pathology and remyelination attempts are impacting neuronal health in ALS is an important aspect in uncovering the role of myelin in the disease.

In our study, we found changes in the oligodendroglial lineage at 10 months in TDP‐43 mice. In the initial characterization of the *TDP‐43* ^*Q331K*^ mouse, motor neuron pathology is identified as early as 2 months (Arnold et al. 2013), suggesting that oligodendrocyte dysfunction in this model does not explicitly cause initial motor neuronal deficits. In contrast, *SOD1* mouse studies have shown increased OPC proliferation and differentiation presymptomatically (Kang et al. 2013; Philips et al. 2013), thus oligodendroglia may indeed be key contributors to ALS initiation and progression in *SOD1* mice. Interestingly, 10 months in the *TDP‐43* ^*Q331K*^ mouse is when astrogliosis and microgliosis occur (Arnold et al. 2013), whereas in *SOD1* models both microglia and astrocytes exhibit presymptomatic reactivity and altered functionality (Gerber et al. 2012; Gomes et al. 2019; Miller et al. 2017), mirroring our oligodendroglia observations. It should also be noted that in the *SOD1* ^*G93A*^ mouse model utilized by Kang et al. (2013), there is a 17‐fold expression of the human mutant SOD1 protein in the diseased mice (Jonsson et al. 2006), compared to a 2‐fold expression of TDP‐43 protein in the *TDP‐43* ^*Q331K*^ mouse (Arnold et al. 2013). Further, while the *TDP‐43* ^*Q331K*^ mouse exhibits TDP‐43 proteinopathy, this mouse lacks gross TDP‐43 aggregation and has no survival deficit, raising the question as to whether oligodendrocyte and myelin changes would be more abundant in a more severe TDP‐43 model of ALS. In the double transgenic *TDP‐43* ^*Q331K/WT*^ mouse model, which has a survival of around 10 weeks, myelin abnormalities in the lumbar spinal cord motor axon roots were noted at 8 weeks of age, suggestive of myelin degeneration (Mitchell et al. 2015); however, further explorations into oligodendroglial behaviors and myelin dysfunction were not conducted, nor was examining the myelin at presymptomatic timepoints. In the *hTDP‐43ΔNLS* transgenic mouse, pathological mislocalization and cytoplasmic accumulation of nuclear TDP‐43 cause progressive motor neuron loss, denervation, muscle and brain mass reduction, motor phenotypes, and early death (Walker et al. 2015). Interestingly, the pathology inducing nuclear mislocalization of TDP‐43 can be suppressed via doxycycline administration and, when done so, mice experience an almost complete functional recovery (Walker et al. 2015). Proteomic analysis of these mice showed a gradual increase in markers of oligodendrogenesis and myelin production both during the later stages of disease and during the “recovery” period (San Gil et al. 2024), suggesting that the reactivity of the oligodendroglia may be contributing to pathology but also may be important for recovery in a more severe model of ALS. It remains unclear precisely how changes to the TDP‐43 protein and its function drive oligodendrocyte and myelin dysfunction in ALS, but given the consistent effects noted in the varied TDP‐43 mouse models, it certainly warrants further investigation.

To our knowledge, we are the first to extensively characterize the cellular and myelinating dynamics of oligodendroglia in a TDP‐43 model of ALS, finding significant changes to the oligodendrocyte lineage and dysfunctional myelin in 10‐month‐old mice. These findings suggest that oligodendroglia and myelin do indeed play a role in ALS pathology. Whether oligodendroglia are intrinsically pathological or are reacting to neuronal deficits remains unknown but does not diminish the concept that preserving oligodendrocyte function may have the capacity to rescue some neuronal function, highlighting the need for further investigation into whether preserving oligodendroglia function could alleviate neuronal pathology in ALS.

## Author Contributions

K.N.L., S.K.B. and D.G.G. conceived the study. K.N.L., S.K.B, J.M., A.K.W., B.J.T. and D.G.G. designed the experiments. K.N.L., G.A.C., D.T. and B.C. performed the experiments. K.N.L. analysed the data. K.N.L. and S.K.B. wrote the paper. All authors have read and agreed to the published version of the manuscript.

## Ethics Statement

All mouse procedures were approved by the Florey Institute of Neuroscience and Mental Health (Ethics number: 20–010‐FINMH). All procedures followed the Australian Code of Practice for the Care and Use of Animals for Scientific Purposes. All animal experiments adhered to the Australian National Health and Medical Research Council published Code of Practice and were approved by the Florey Institute of Neuroscience and Mental Health Animal Ethics Committee (Ethics number: 20–010‐FINMH).

## Conflicts of Interest

The authors declare no conflicts of interest.

## Supporting information


**Figure S1:** Animal weights and behavior. (A) There was no change in the average weights between WT and TDP‐43 animals, however weight did increase over time (2‐Way ANOVA *p* ≥ 0.0001). (B) Rotarod Walking Test analyses showed a significant decrease in latency to fall for TDP‐43 mice compared to WT (2‐way ANOVA *p* = 0.0416). Post hoc analyses showed significant decreases in TDP‐43 mice rotarod performance between 2 and 3 months (*p* = 0.0104), between 9 and 10 months (*p* = 0.0208), and at 10 months (*p* = 0.0372). (C) Rotarod comparison at 10 months also shows a significant decrease for the TDP‐43 mice compared to WT (*p* = 0.0094). (D) Hind‐Limb Grip Strength analysis revealed no significant difference between WT and TDP‐43 mice (2‐way ANOVA *p* = 0.3672) when comparing longitudinally, however direct comparisons at 10 moths show a significant reduction in grip strength in TDP‐43 mice compared to WT ((E); *p* = 0.0031). All data are presented as mean ± S.E.M, *p ≤ 0.05, **p ≤ 0.01; longitudinal data analyzed via 2‐way ANOVA with Tukey's post hoc test; 10 months comparison via Student's unpaired *t* tests; *n* = 7–8 mice per genotype.
**Figure S2:** Oligodendrocyte precursor cell (OPC) cell cycle dynamics at P15, P30, 3 and 5 months using double‐s phase labelling. (A) Representative images of stained mouse lumbar spinal cord tissue with DAPI (blue) labelling all nucleated cells, BrdU (green) labelling cells injected 2.5 h prior to culling, EdU (magenta) labelling cells injected 0.5 h prior to culling, and PDGFRα (red) labelling OPCs. OPC S‐phase length was not changed between WT and TDP‐43 mice in the dorsal column (DC) of the lumber spinal cord in mice aged P15 and P30 (B). No changes were found in the s‐phase length of 3 and 5 months mice in the corticospinal tract (CST; C), ventral horn gray matter (VHGM; D), or ventral horn white matter (VHWM; E). Similarly, no changes to the cell cycle length were found at P15 and P30 in the DC (F), not at 3 and 5 months in the CST (G), VHGM (H), or VHWM (I). All data are presented as mean ± S.E.M, *p ≤ 0.05; 2‐way ANOVA with Tukey's post hoc test; *n* = 3 mice per genotype per age.
**Figure S3:** Oligodendrocyte precursor cell (OPC) density and growth fraction of OPCS at P15 and P30. There were no changes in OPC density at either P15 or P30 in the dorsal column (DC) of the lumbar spinal cord of WT and TDP‐43 mice (A), nor any changes in the growth fraction (B). All data are presented as mean ± S.E.M, *p ≤ 0.05; 2‐way ANOVA with Tukey's post hoc test; *n* = 6 mice per genotype per age.
**Figure S4:** Densities of mature oligodendrocytes. (A) Representative image of mature oligodendroglia (CC1; green), all oligodendroglia (Olig2; red), and all nucleated cells (DAPI; blue). Scale bars = 200 μm. (B) There was trend for an increase in mature oligodendroglia density in the ventral horn gray matter (VHGM) of TDP‐43 mice compared to WT at 10 months (2‐way ANOVA *p* = 0.0126; post hoc at 10 months *p* = 0.0876) with no change at 8 months. There were no changes in the ventral horn white matter (VHWM; C) or the corticospinal tract (CST; D) All data are presented as mean ± S.E.M, *p ≤ 0.05; 2‐way ANOVA with Tukey's post hoc test; *n* = 3–4 per genotype per age.
**Figure S5:** Interneuronal pathology at 10 months. Representative images of the ventral horn gray matter of 10 months WT and TDP‐43 mice to show myelinated interneurons (parvalbumin; red), apoptotic cells (caspase 3; green), and nucleated cells (DAPI; blue). Scale bars = 20 μm.
**Figure S6:** Myelin analysis via Transmission Electron Microscopy. (A) The frequency of axon diameters were not altered between WT and TDP‐43 mice, bin width 0.5 μm. (B) The g‐ratios compared to axon diameter were not difference between WT and TDP‐43 mice, bin width 0.5 μm. (C) The density of myelinated fibers was unchanged between TDP‐43 and WT mice at 10 months in the dorsal column. All data are presented as mean ± S.E.M, *p ≤ 0.05, multiple unpaired *t* tests and student's unpaired *t* test, *n* = 3 animals per genotype, ≥ 180 axons analyzed per animal.

## Data Availability

The data that support the findings of this study are available from the corresponding author upon reasonable request.
