# Supplementary material for: Oligodendroglial Densities and Myelin Structure Are Altered in TDP‐43 Related Amyotrophic Lateral Sclerosis
Source: Glia. 2025 Oct 10;74(1):e70090. doi: 10.1002/glia.70090 (PMC12667226; doi:10.1002/glia.70090)
Supplement: Supplementary file 1 — Figure S1: Animal weights and behavior. (A) There was no change in the average weights between WT and TDP‐43 animals, however weight did increase over time (2‐Way ANOVA p ≥ 0.0001). (B) Rotarod Walking Test analyses showed a significant decrease in latency to fall for TDP‐43 mice compared to WT (2‐way ANOVA p = 0.0416). Post hoc analyses showed significant decreases in TDP‐43 mice rotarod performance between 2 and 3 months (p = 0.0104), between 9 and 10 months (p = 0.0208), and at 10 months (p = 0.0372). (C) Rotarod comparison at 10 months also shows a significant decrease for the TDP‐43 mice compared to WT (p = 0.0094). (D) Hind‐Limb Grip Strength analysis revealed no significant difference between WT and TDP‐43 mice (2‐way ANOVA p = 0.3672) when comparing longitudinally, however direct comparisons at 10 moths show a significant reduction in grip strength in TDP‐43 mice compared to WT ((E); p = 0.0031). All data are presented as mean ± S.E.M, *p ≤ 0.05, **p ≤ 0.01; longitudinal data analyzed via 2‐way ANOVA with Tukey's post hoc test; 10 months comparison via Student's unpaired t tests; n = 7–8 mice per genotype. Figure S2: Oligodendrocyte precursor cell (OPC) cell cycle dynamics at P15, P30, 3 and 5 months using double‐s phase labelling. (A) Representative images of stained mouse lumbar spinal cord tissue with DAPI (blue) labelling all nucleated cells, BrdU (green) labelling cells injected 2.5 h prior to culling, EdU (magenta) labelling cells injected 0.5 h prior to culling, and PDGFRα (red) labelling OPCs. OPC S‐phase length was not changed between WT and TDP‐43 mice in the dorsal column (DC) of the lumber spinal cord in mice aged P15 and P30 (B). No changes were found in the s‐phase length of 3 and 5 months mice in the corticospinal tract (CST; C), ventral horn gray matter (VHGM; D), or ventral horn white matter (VHWM; E). Similarly, no changes to the cell cycle length were found at P15 and P30 in the DC (F), not at 3 and 5 months in the CST (G), VHGM (H), or VH [file GLIA-74-0-s001.docx]

Oligodendroglial Densities and Myelin Structure are Altered in TDP-43 Related Amyotrophic Lateral Sclerosis

Running Head: Oligodendroglial Alterations in ALS

Katherine Lewis^1^, Georgina A Craig^2,^, Joel Mason^1^, Doris Tomas^1^, Brittany Cuic^1^, Adam K Walker^3,4^, David Gonsalvez^5^, Bradley Turner^1^ & Samantha Barton^1^

1. The Florey Institute of Neuroscience and Mental Health, Melbourne VIC, Australia
2. Li Ka Shing Knowledge Institute, Unity Health, St Michael's Hospital, Toronto, Canada
3. Clem Jones Centre for Ageing Dementia Research, Queensland Brain Institute, Brisbane QLD, Australia
4. Sydney Pharmacy School, Faculty of Medicine and Health, The University of Sydney
5. Department of Anatomy and Developmental Biology, Monash University, Melbourne VIC, Australia

**Supplementary Figure 1**. Animal Weights and Behaviour. **A**. There was no change in the average weights between WT and TDP-43 animals, however weight did increase over time (2-Way ANOVA p > 0.0001). **B.** Rotarod Walking Test analyses showed a significant decrease in latency to fall for TDP-43 mice compared to WT (2-way ANOVA p = 0.0416). *Post hoc* analyses showed significant decreases in TDP-43 mice rotarod performance between 2-3 months (p = 0.0104), between 9 and 10 months (p = 0.0208), and at 10 months (p = 0.0372). **C.** Rotarod comparison at 10 mo also shows a significant decrease for the TDP-43 mice compared to WT (p = 0.0094). **D.** Hind-Limb Grip Strength analysis revealed no significant difference between WT and TDP-43 mice (2-way ANOVA p = 0.3672) when comparing longitudinally, however direct comparisons at 10 mo show a significant reduction in grip strength in TDP-43 mice compared to WT (**E**.; p = 0.0031). All data are presented as mean ± S.E.M, *p < 0.05, **p < 0.01; longitudinal data analysed via 2-way ANOVA with Tukey’s *post hoc* test; 10 mo comparison via Student’s unpaired t-tests; n = 7-8 mice per genotype.

**Supplementary Figure 2.** Oligodendrocyte precursor cell (OPC) cell cycle dynamics at P15, P30, 3 mo and 5 mo using double-s phase labelling. **A**. Representative images of stained mouse lumbar spinal cord tissue with DAPI (blue) labelling all nucleated cells, BrdU (green) labelling cells injected 2.5 hr prior to culling, EdU (magenta) labelling cells injected 0.5 hr prior to culling, and PDGFRα (red) labelling OPCs. OPC S-phase length was not changed between WT and TDP-43 mice in the dorsal column (DC) of the lumber spinal cord in mice aged P15 and P30 (**B**). No changes were found in the s-phase length of 3 mo and 5 mo mice in the corticospinal tract (CST; **C**), ventral horn grey matter (VHGM; **D**), or ventral horn white matter (VHWM; **E**). Similarly, no changes to the cell cycle length were found at P15 and P30 in the DC (**F**), not at 3 mo and 5 mo in the CST (**G**), VHGM (**H**), or VHWM (**I**). All data are presented as mean ± S.E.M, *p < 0.05; 2-way ANOVA with Tukey’s *post hoc* test; n = 3 mice per genotype per age.

.


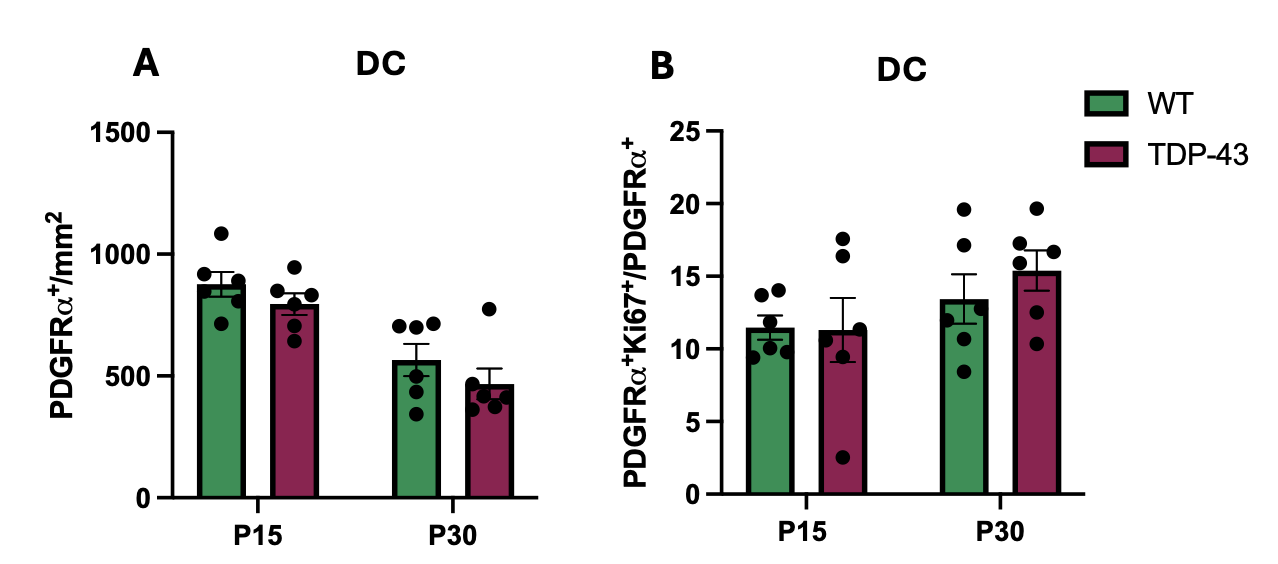


**Supplementary Figure 3**. Oligodendrocyte precursor cell (OPC) density and growth fraction of OPCS at P15 and P30. There were no changes in OPC density at either P15 or P30 in the dorsal column (DC) of the lumbar spinal cord of WT and TDP-43 mice (**A**), nor any changes in the growth fraction (**B**). All data are presented as mean ± S.E.M, *p < 0.05; 2-way ANOVA with Tukey’s *post hoc* test; n = 6 mice per genotype per age.

**Supplementary Figure 4.** Densities of mature oligodendrocytes. **A.** Representative image of mature oligodendroglia (CC1; green), all oligodendroglia (Olig2; red), and all nucleated cells (DAPI; blue). Scale bars = 200µm **B.** There was trend for an increase in mature oligodendroglia density in the ventral horn grey matter (VHGM) of TDP-43 mice compared to WT at 10 mo (2-way ANOVA p = 0.0126; *post* hoc at 10 mo p = 0.0876) with no change at 8 mo. There were no changes in the ventral horn white matter (VHWM; **C.**) or the corticospinal tract (CST; **D.**) All data are presented as mean ± S.E.M, *p < 0.05; 2-way ANOVA with Tukey’s *post hoc* test; n = 3-4 per genotype per age.


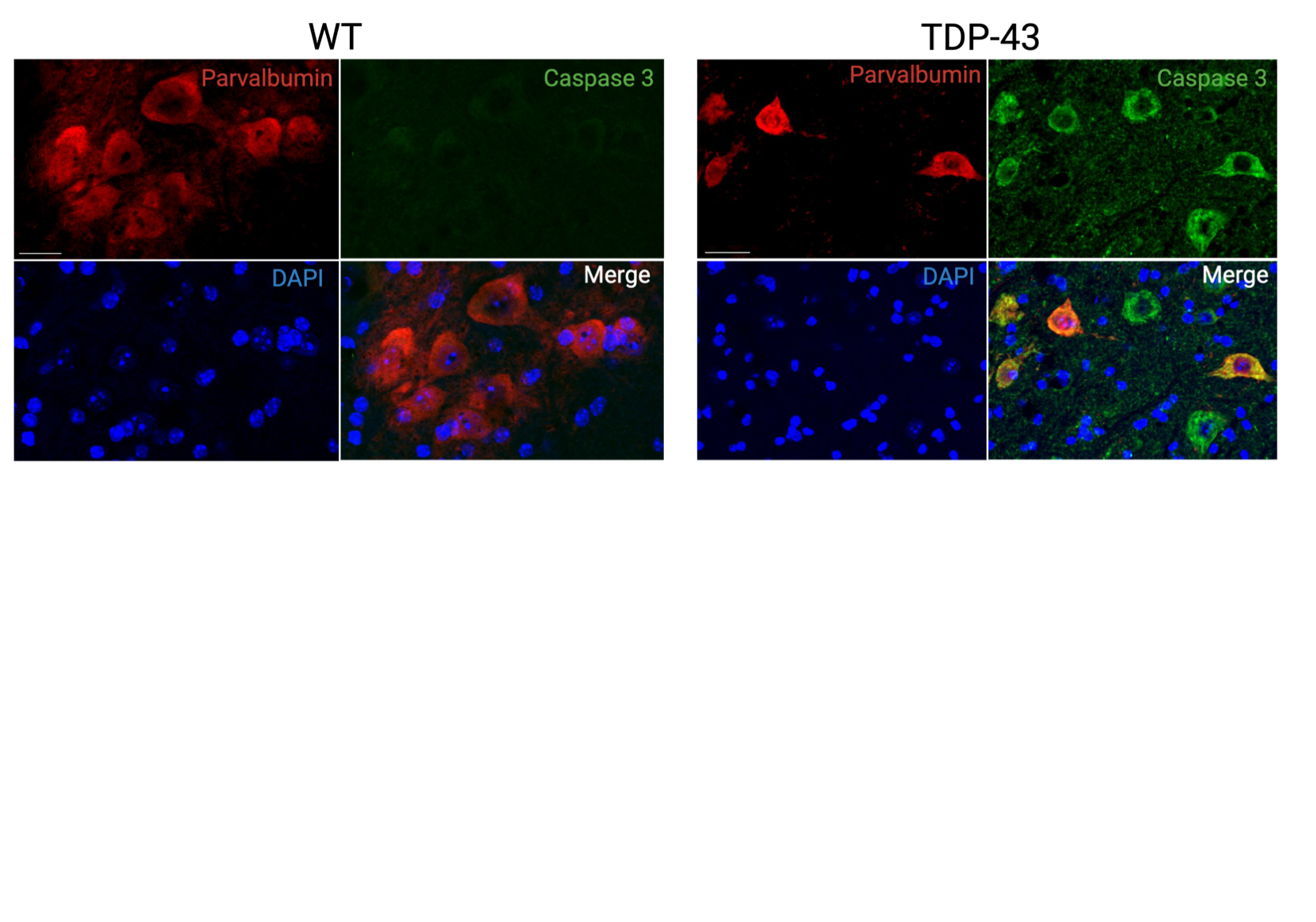


**Supplementary Figure 5.** Interneuronal pathology at 10 mo. Representative images of the ventral horn grey matter of 10 mo WT and TDP-43 mice to show myelinated interneurons (parvalbumin; red), apoptotic cells (caspase 3; green), and nucleated cells (DAPI; blue). Scale bars = 20µm.

**Supplementary Figure 6.** Myelin analysis via Transmission Electron Microscopy. **A.** The frequency of axon diameters were not altered between WT and TDP-43 mice, bin width 0.5 µm. **B.** The g-ratios compared to axon diameter were not difference between WT and TDP-43 mice, bin width 0.5µm. **C.** The density of myelinated fibres was unchanged between TDP-43 and WT mice at 10 mo in the dorsal column. All data are presented as mean ± S.E.M, *p < 0.05, multiple unpaired t-tests and student’s unpaired t-test, n = 3 animals per genotype, >180 axons analysed per animal.
